# Synthesis of Polycarboxylate Viscosity Reducer and the Effect of Different Chain Lengths of Polyether on Viscosity Reduction of Heavy Oil

**DOI:** 10.3390/polym14163367

**Published:** 2022-08-18

**Authors:** Junqi Wang, Ruiqing Liu, Yiwen Tang, Junfeng Zhu, Yonghui Sun, Guanghua Zhang

**Affiliations:** 1 The Key Laboratory of Well Stability and Fluid & Rock Mechanics in Oil and Gas Reservoir of Shaanxi Province, Xi’an Shiyou University, Xi’an 710065, China; 2 Shaanxi Key Laboratory of Chemical Additives for Industry, College of Chemistry and Chemical Engineering, Shaanxi University of Science and Technology, Xi’an 710021, China

**Keywords:** polyether-type polycarboxylic acid, viscosity reduction, viscosity reducer, heavy oil

## Abstract

Since there are not many studies on the application of polymeric surfactants in viscosity reduction emulsification of heavy oil, a series of polyether carboxylic acid–sulfonate polymeric surfactants were synthesized. The viscosity reduction performance and the effect of different chain lengths on the viscosity reduction effect were also investigated. The viscosity reduction, emulsification, wetting, and foaming performance tests showed that the viscosity reduction performance of this series of polymeric surfactants was excellent, with the viscosity reduction rate exceeding 95%, and the viscosity was reduced to 97 mPa·s by the polymeric surfactant with a molecular weight of 600 polyethers. It was also concluded that among the three surfactants with different side chains, the polymeric surfactant with a polyether molecular weight of 600, which is the medium side-chain length, had the best viscosity reduction performance. The study showed that the polyether carboxylic acid–sulfonate polymer surfactant had a promising application in the viscosity reduction of heavy oil.

## 1. Introduction

With the continuous exploitation of global oil resources, oil extraction has entered the tertiary stage to extract heavy and ultraheavy oil. As a result of surveys, the global share of heavy and superheavy oil accounts for 40% of proven reserves, exceeding that of conventional light oil reserves [1,2]. Moreover, globally, the recoverable resources of heavy oil account for 29% of unconventional oil and gas [3]. Still, heavy oil has the characteristics of a high viscosity, high density, and difficult flow, which makes it challenging to extract heavy oil. Therefore, reducing the viscosity of heavy oil and increasing its flowability is the key to improving the recovery rate of heavy oil. The reasons for the high viscosity of heavy oil are the presence of gums and asphaltenes in heavy oil. Moreover, gums and asphaltenes form large aggregates under the influence of hydrogen bonding, π-π stacking, electrostatic force, metal complexes, side-chain length, etc. [4,5,6].

The current methods to reduce the viscosity of heavy oil are mainly heating, dilution, and emulsification. However, heating technology has a high energy consumption and causes safety problems; dilution technology reduces the physical properties of heavy oil [7]. Therefore, emulsification has become a significant option for heavy-oil extraction. Emulsification is mainly achieved by adding chemical oil repellents, i.e., chemical drive, including polymer drive, alkali drive, surfactant drive, ternary complex drive, and polymer surfactant drive [8]. In contrast, polymer surfactant drive has the excellent properties of both polymer and surfactant: a high viscosity, outstanding dispersibility, and film formation, which avoids the possible undesirable effects of low molecular surfactants and high molecular compounds when used in combinations [9], such as the compound system separating into two phases during the flow process, resulting in the loss of surfactant to the surface of the formation through adsorption [10]. In addition, the presence of strong bases in the ternary complex drive system has a detrimental effect on polymer properties [10]. In contrast, most polymeric surfactants are not only more stable but also biocompatible and environmentally friendly [9]. Therefore, polymeric surfactants can have a wide range of applications for viscosity reduction in heavy oil.

At present, the ability of most polymeric surfactants to reduce oil–water interfacial tension is not significant enough. Moreover, most scholars believe that the ability to reduce interfacial tension is the main criterion for evaluating whether the surfactant can be used in oil repelling [10]. Therefore, there are not many studies on the application of polymeric surfactants to oil repelling. However, to analyze and study the viscosity reduction performance and emulsification performance of heavy oil, some scholars have studied the application of polyether carboxylic acid–sulfonic acid viscosity reducing agent. Qin Bing et al. found that the viscosity reduction rate of a polyether carboxylic acid–sulfonic acid copolymer could still reach 90% under high temperature and high mineralization. Its viscosity reduction effect was better than the viscosity reduction effect of sulfonate or phosphate emulsification viscosity reducers. The study showed that the cocondensation had both hydrophilic groups sulfonic acid groups, carboxylic acid groups, and oxyethylene groups, and the relative molecular mass was larger and more polar. Therefore, the “compensating” effect of different anionic groups made it easier to replace asphaltenes and other substances from the oil–water interface, forming an active water film and completing the transformation of emulsions [11]. Xiao Sa, Sun Yongtao et al. also showed that the viscosity reduction rate of polyether carboxylates for heavy oil could reach more than 95%, and the viscosity could be reduced to less than 100 mPa·s [12]. Bao Xinning et al. showed that a coconut oil polyether carboxylate anionic repellent had good oil-repellent performance [13]. Zhiwei Wu et al. showed that a polymeric surfactant had a good viscosity reduction and emulsification performance for heavy oil [14]. Therefore, it is promising to use polyether carboxylic acid sulfonates as viscosity-reducing agents for the emulsification and viscosity reduction of heavy oil.

Based on the above reasons, in the present study, we synthesized a new polymeric surfactant with acrylic acid (AA), sodium p-styrenesulfonate (SSS), and allyl polyoxyethylene ether (APEG) analyzed its viscosity-reducing effect on heavy oil, proposed an explanation for its mechanism, analyzed the impact of different side-chain lengths of this polymeric surfactant on the viscosity-reducing effect, and also optimized the reaction conditions.

## 2. Materials and Methods

### 2.1. Materials

Sodium p-styrenesulfonate (SSS) (Aladdin, Hong Kong, China); acrylic acid (AA) (Tianjin Damao Chemical Reagent Factory, Tianjin, China); potassium persulfate, sodium bisulfite (Tianjin Damao Chemical Reagent Factory); allyl polyoxyethylene ether series (APEG, Jiangsu Haian Petrochemical Factory, Nantong, China); sodium hydroxide (Tianjin Damao Chemical Reagent Factory); dichloromethane (Tianjin Damao Chemical Reagent Factory); deuterated water (Shanghai Maclean Biochemistry, Shanghai, China); all were analytically pure; spectrally pure potassium bromide (Tianjin Damao Chemical Reagent Factory); superior pure sodium nitrate (Chengdu Kolon Chemical Co., Chengdu, China).

### 2.2. Synthesis of Polyether Carboxylic Acid–Sulfonate Polymeric Surfactants

**Figure 1:**
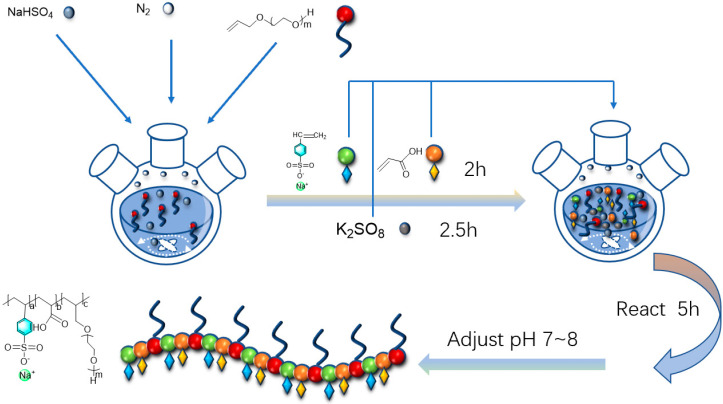
Reaction steps diagram.

**Figure 2:**
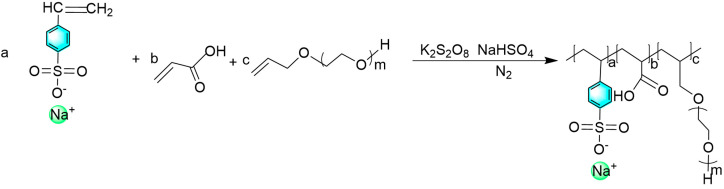
Reaction equations.

Allyl polyoxyethylene ethers (APEG315, APEG600, APEG1200) of different relative molecular masses were reacted with acrylic acid (AA) and sodium p-styrenesulfonate (SSS) in the molar ratio of 1:4:1 to obtain a series of polyether carboxylic acids with different side-chain lengths, SAAP315, SAAP600, and SAAP1200. The total weight of the monomer was 30 g, and that of the water was 70 g. Sodium bisulfite was 1% of the total weight of the monomer, and potassium persulfate was 3% of the total weight of the monomer. A certain amount of distilled water was added, and the solution was stirred with a glass rod to fully dissolve potassium persulfate, sodium bisulfite, allyl polyoxyethylene ether, and sodium p-styrenesulfonate. Then, we added sodium bisulfite and allyl polyoxyethylene ether to a three-necked flask equipped with a reflux condenser and a stirring magnet. Sodium p-styrenesulfonate, potassium persulfate, and acrylic acid were then added to a constant pressure funnel in a certain ratio. The oil bath was heated, and nitrogen gas was introduced to replace the air in the flasks, and the temperature was raised to 80 °C. The nitrogen gas was stopped, and sodium p-styrenesulfonate, acrylic acid, and potassium persulfate were added dropwise for 2 h, 2 h, and 2.5 h, respectively, and the reaction was terminated after 5 h. After the reaction products were cooled to room temperature, the pH was adjusted at 7–8 with a 20% sodium hydroxide solution. The polyether carboxylic acid products with different side-chain lengths were obtained [15]. The reaction steps shown in Figure 1 and reaction equations shown in Figure 2 were as follows:

The reaction products were:

Polyether polysulfonic acid carboxylate 315 (SAAP315);

Polyether polysulfonic acid carboxylate 600 (SAAP600);

Polyether polysulfonic acid carboxylate 1200 (SAAP1200).

### 2.3. Characterization

The samples were tested with an EQUI NX55 infrared spectrometer from Brucher, Germany. The potassium bromide was dried in an oven at 100 °C. The polyethercarboxylic acid–sulfonate polymer surfactant was dried, then finely ground with an agate mortar and kept dry in a drying container and set aside. A small amount of potassium bromide and polyether carboxylic acid–sulfonate products were prepared by the press method to make them translucent with 90% light transmission. The test wave number range was 50–4000 cm^−1^.

The dried polyether carboxylic acid–sulfonate series products were characterized and tested by using a Bruker Varian Inova 500 NB NMR hydrogen spectrometer with 5 mg dissolved in 10 mL of deuterated water in a 600 MHz NMR system.

For the gel permeation chromatography (Waters Model 2414-515, Malvern Instruments, Malvern, UK) test, 0.1 mol/L of analytically pure sodium nitrate aqueous solution was used as the mobile phase. The detector was a diode array detector (DAD). Polyethylene glycol was used as the standard sample, and the sample was prepared as a dilute solution of 3 mg/mL. The determination was carried out at a constant temperature of 30 °C, a flow rate of 1 mL/min, and an injection volume of 20 μL.

### 2.4. Determination of Heavy Oil Properties

The heavy oil fraction was determined by GC-MS (7890B-5977A Agilent Technologies, Santa Clara, CA, USA) using dichloromethane as a solvent.

### 2.5. Viscosity-Reducing Properties of Polyether Carboxylic Acid–Sulfonate Series

The North China heavy oil was measured using a digital display viscometer produced by Brookfield Co. The 5% polyether carboxylic acid–sulfonate solution was mixed with North China heavy oil in the ratio of 1:1 by volume, heated at a water bath temperature of 50 °C for 15 min, and stirred with an emulsifier (D-160) at a speed of 8000 r/min for 4 min. The emulsion layer viscosity was measured at that temperature after standing for 1 min. At the same time, an equal volume of heavy oil was taken under the same conditions to determine its viscosity as a control group.

### 2.6. Surface Tension Measurement

The surface tension of the polyether carboxylic acid series was determined by using the DCAT21 surface interfacial tension meter. Solutions of different mass concentrations were configured separately, and the surface tension was determined by the platinum sheet method at room temperature.

### 2.7. Contact Angle Measurement

The contact angle of the polyether carboxylic acid series was measured by a contact angle meter of German Orient Delphi. North China heavy oil was applied evenly onto the slide. Then, solutions of different mass concentrations were configured separately. The contact angle of the droplets of the sample solution dropping onto the surface of the heavy oil was measured at room temperature using the contact angle meter.

### 2.8. Emulsification Speed Measurement

The solutions of different mass concentrations were configured separately and mixed with North China heavy oil in the ratio of 1:1. Heated at a water bath temperature of 50 °C for 15 min, then stirred with an emulsifier at a speed of 8000 r/min for 2.5 min. Finally, the volume of the emulsion was measured at a resting time of 1 min.

$$\mathrm{Emulsification}\;\mathrm{speed}=\frac{\mathrm{emulsion}\;\mathrm{volume}}{\mathrm{stirring}\;\mathrm{time}}$$

where the emulsion volume is in milliliters, and the stirring time is in minutes.

### 2.9. Stability of Emulsions

The solutions of different mass concentrations were mixed with North China heavy oil in the ratio of 1:1, heated at a water bath temperature of 50 °C for 15 min, then stirred for 2.5 min with an emulsifier at a speed of 8000 r/min and the content of precipitated water after standing for 10 min was measured.

$$\mathrm{Water}\;\mathrm{separation}\;\mathrm{rate}=\frac{\;\mathrm{volume}\;\mathrm{of}\;\mathrm{precipitated}\;\mathrm{water}}{\left(\mathrm{emulsion}\;\mathrm{volume}\;+\;\mathrm{volume}\;\mathrm{of}\;\mathrm{precipitated}\;\mathrm{water}\right)\;}\;\times \;100\%$$

### 2.10. Foaming Performance and Foam Stability

Solutions of different mass concentrations were configured separately and stirred with an emulsifier at 8000 r/min for 10 s. The foam heights were measured just after stirring was stopped and at 5 min, respectively. The maximum foam height at the stop of the stirring reflected the foaming ability of the polyether carboxylic acid-sulfonate series. The foam height at 5 min reflected the foam stability [16,17].

## 3. Results and Discussion

### 3.1. Characterization

#### 3.1.1. FT-IR Spectroscopy

**Figure 3:**
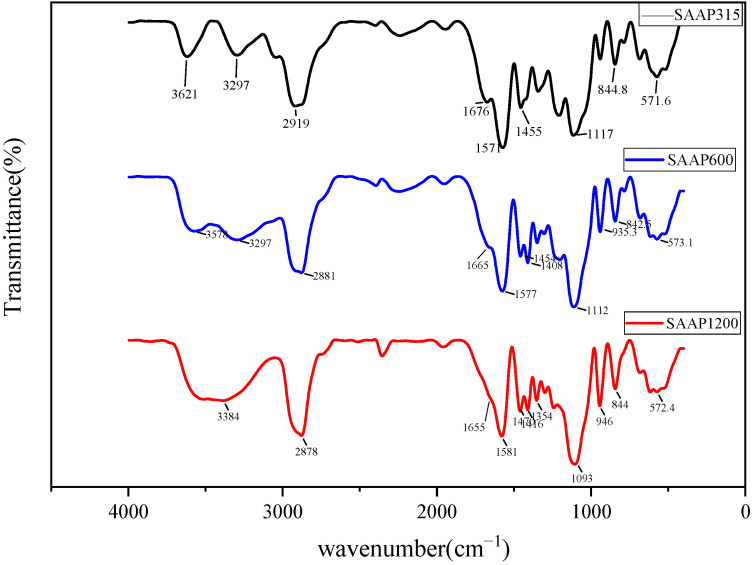
Infrared spectra of products resulting from the reaction of three different APEGs as monomers.

From Figure 3, it can be seen that all three different products have broad absorption peaks near 3300 cm^−1^, which is the stretching vibration of -OH of the carboxyl group; all have strong absorption peaks near 2900 cm^−^^1^, which is the stretching vibration of saturated C-H bond; all have absorption peaks of C=O near 1700 cm^−1^. There are small absorption peaks near 1600 cm^−1^, presumably some C=C absorption peaks, indicating that most of the C=C double bonds completely reacted, and the reaction was more adequate. There are skeletal vibration peaks of a benzene ring near 1580 cm^−1^ and 1450 cm^−1^. Strong absorption peaks near 1100 cm^−1^ are judged to be the absorption peaks of C-O-C, and the bending vibration characteristic peak of COOH was observed near 930 cm^−1^. The peak near 840 cm^−1^ was the para-disubstitution absorption peak of a benzene ring. In summary, the reaction product is the target product.

#### 3.1.2. ^1^HNMR

**Figure 4:**
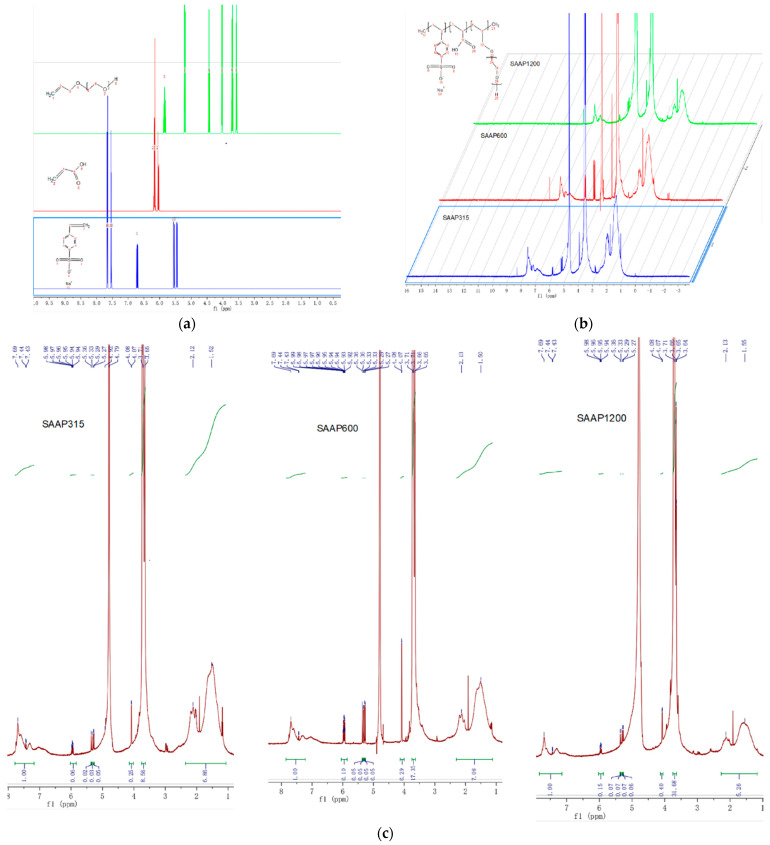
(**a**) ^1^HNMR prediction of the reaction monomer; (**b**) ^1^HNMR images of products made from the reaction of three different APEGs as monomers. (**c**) Integration of ^1^HNMR images of products.

In Figure 4, a comparison of the NMR predictions of the reaction monomer with the actual NMR spectra of the three reaction products shows that all three products have two peaks of the para-disubstituted benzene ring near δ = 7.4 and 7.7 ppm. There are no absorption peaks near δ = 5.5 and 6.7 ppm, indicating the complete reaction of the C=C double bond of SSS. Similarly, there are no absorption peaks near δ = 6.0, 6.2 ppm, suggesting a complete C=C double bond reaction of AA. There are faint absorption peaks near δ = 5.3 and 5.9 ppm with a small integrated area, indicating that most of the C=C double bonds of APEG completely reacted. There are two peaks of C of APEG near the C=C double bond near δ = 4 ppm and two peaks of the -CH_2_-CH_2_-O-group of APEG near δ = 3.5–3.7 ppm. There are peaks of H of the main chain of the product between δ = 1~2 ppm. Meanwhile, taking the integral area of H (δ = 7.4, 7.7 ppm) on the benzene ring of SSS as 1, the integral area of H (δ = 3.5–3.7 ppm) of the -CH2-CH2-O-group of APEG_n_ (n = 315, 600, 1200) for the three products is approximated as 7:14:28. This means that the molar ratio SSS/APEG = 1:1. The integrated areas of H of -CH2- and -CH- near δ = 1~2 ppm are all close to 6, indicating that the molar ratio AA/SSS/APEG = 4:1:1. From the above analysis, the reaction product should be the target product, and based on the ratio of the integral area of H (δ = 7.4, 7.7 ppm) on the benzene ring of SSS and the C=C double bond H (δ = 5.3, 5.9 ppm) of unreacted APEG, it can be calculated that the purity of SAAP315 is roughly 80%, that of SAAP600 is around 60% and that of SAAP1200 is approximately 50%.

#### 3.1.3. Relative Molecular Mass Distribution

Table 1 shows the average relative molecular mass of three different APEG as monomer reaction products. According to the data in the table, we can see that the heavy average molecular weight of the three viscosity reducers is more than 40,000; SAAP315 and SAAP600′s heavy average molecular weight was more than 50,000, higher than that of SAAP1200. The highest average molecular weight was that of SAAP315. Overall, the greater the relative molecular mass of the viscosity reducers, the more adsorption on the surface of heavy oil, the stronger the emulsification capacity of heavy oil, and the better the viscosity reduction effect.

### 3.2. Determination of Heavy Oil Properties

The main components of North China heavy oil were identified by GC-MS as shown in Table 2.

The components of the heavy oil are very complex, especially the heavy components asphaltenes and gums. A typical heavy fraction model includes three asphaltenes and six gums. The light component model includes a certain proportion of alkanes (hexane, heptane, octane, nonane), cycloalkanes (cyclohexane, cycloheptane), and aromatic hydrocarbons (benzene, toluene). The mass fraction of gums and asphaltenes is 38% [18,19,20,21,22].

### 3.3. Viscosity-Reducing Properties of Polyether Carboxylic Acid–Sulfonate Viscosity Reducers

**Figure 5:**
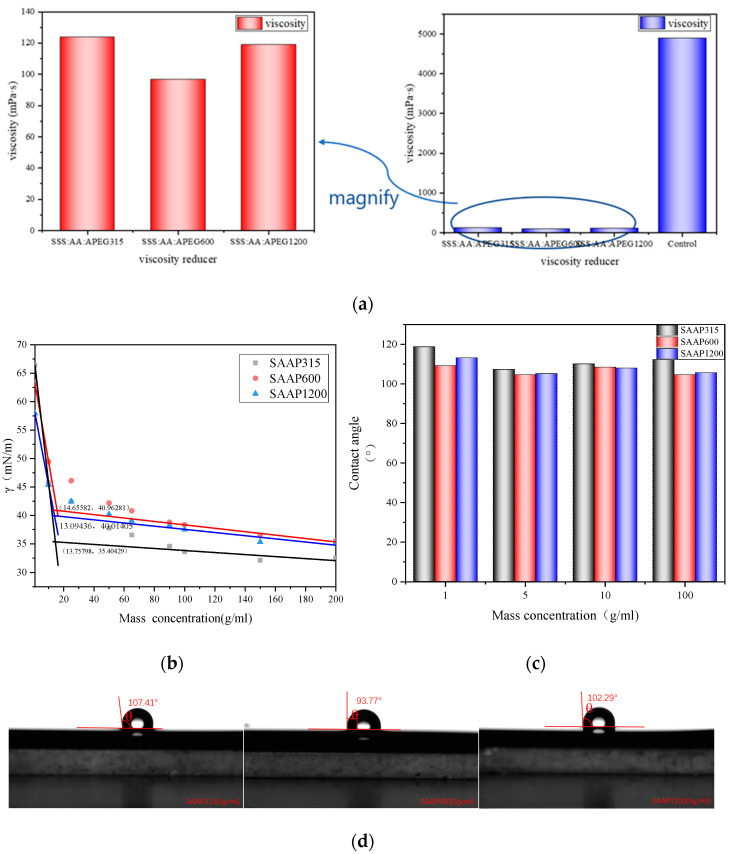
(**a**)The viscosity of different viscosity-reducer solutions after emulsification with heavy oil. (**b**) Surface tension diagram of three different viscosity reducers. (**c**) Contact angle values of different viscosity reducers with different concentrations. (**d**) Contact angle pictures of different viscosity reducers at 5 g/mL.

The viscosity of three different viscosity-reducer solutions emulsified with heavy oil is shown in Figure 5a below. It can be seen that all three viscosity-reducer solutions reduce the viscosity of heavy oil significantly, and the viscosity reduction rate reaches more than 95%; among the three viscosity-reducer solutions SAAP600 has the best viscosity reduction effect, with a viscosity directly reduced to 97 mPa·s. This is mainly because the lipophilic side chains and main chains of the viscosity reducers are adsorbed on the surface of heavy oil. In contrast, the hydrophilic groups are adsorbed with water molecules. Therefore, the molecules of viscosity reducers replace the asphaltene molecules initially distributed on the oil–water interface, thus destroying the network structure formed by the asphaltene accumulation at the oil–water interface [23]. Thus, the viscosity reduction effect is achieved. The SAAP600 molecule has long lipophilic side chains, which are more easily adsorbed on the surface of heavy oil, and also has more hydrophilic groups, so it can better replace the asphaltene molecules and destroy the reticulation structure of the oil–water interface to achieve better viscosity reduction effect [24].

### 3.4. Surface Tension of Polyether Carboxylate–Sulfonate Viscosity Reducers

The surface tensions of SAAP315, SAAP600, and SAAP1200 are shown in Figure 5b below. The surface tensions of the viscosity-reducer solutions with different mass concentrations are plotted as scatter plots, and the points in the front and back parts were fitted linearly. The intersection of the two fitted straight lines is the critical micelle concentration (CMC) of the viscosity reducer, and the matched analyzed straight lines show that the CMC of SAAP315 is 13.758 g/mL, the CMC of SAAP600 is 14.656 g/mL, and the CMC of SAAP1200 is 13.094 g/mL. If the conversion is done according to the heavy average molecular weight, the CMC of SAAP315 is 2.3 × 10^−4^ mol/mL, the CMC of SAAP600 is 2.6 × 10^−4^ mol/mL, and the CMC of SAAP1200 is 2.8 × 10^−4^ mol/mL; then, the CMC is ordered as follows: SAAP315 < SAAP600 < SAAP1200. This indicates that SAAP315 is more capable of forming micelles. The γ_CMC_ of SAAP315 is 35.404 mN/m, which is smaller than that of SAAP600 and SAAP1200, meaning that SAAP315 has a more vital ability to reduce surface tension at low concentrations, while SAAP600 and SAAP1200 have similar skills. Because the principle of surface tension reduction by surfactants is that the hydrophilic groups of surfactant molecules are dissolved in water, while the lipophilic groups are in contact with air, the surfactants reduce the surface free energy of water by regular arrangement between the interface of water and air, which results in a reduction of the surface tension [25,26]. Then, it is presumed that it is because the side chains of SAAP600 and SAAP1200 are longer than those of SAAP315, which makes the spatial site resistance between adjacent surfactant molecules larger, and the side chains interfere with each other, which makes SAAP600 and SAAP1200 not able to arrange themselves closely and regularly on the surface of the water. Hence, their ability to reduce surface tension is lower than that of SAAP315.

### 3.5. Contact Angle of Polyether Carboxylic Acid–Sulfonate Viscosity Reducers

The contact angle test data of the solutions formulated with different concentrations of the three viscosity-reducing agents are shown in Figure 5c. The contact angle is a substantial physical quantity to judge the wetting performance of a viscosity-reducer solution on heavy oil. The smaller the contact angle is, the better the wetting performance of the viscosity reducer on heavy oil is, and the more beneficial to the viscosity reduction. The contact angle of the three viscosity reducers on heavy oil is SAAP600 < SAAP1200 < SAAP315, and the contact angle size of the three viscosity reducers on heavy oil with the concentration of the solution tends to decrease first with the increasing concentration, and then increases with the increasing concentration of the solution. These phenomena indicate that SAAP600 has the best wetting performance for heavy oil among the three viscosity reducers. This is because SAAP600 has longer lipophilic polyether side chains compared with SAAP315, which gives it a better affinity with heavy oil than SAAP315. At the same time, because the heavy average molecular weight of SAAP600 is higher than that of SAAP1200, it has more polyether side chains inside the molecule, resulting in a better affinity for heavy oil, so SAAP600 has the smallest contact angle [27,28]. The measured contact angle of different viscosity reducers at 5 g/mL is shown in Figure 5d below.

### 3.6. Emulsification Speed of Polyether Carboxylic Acid–Sulfonate Viscosity Reducers

**Figure 6:**
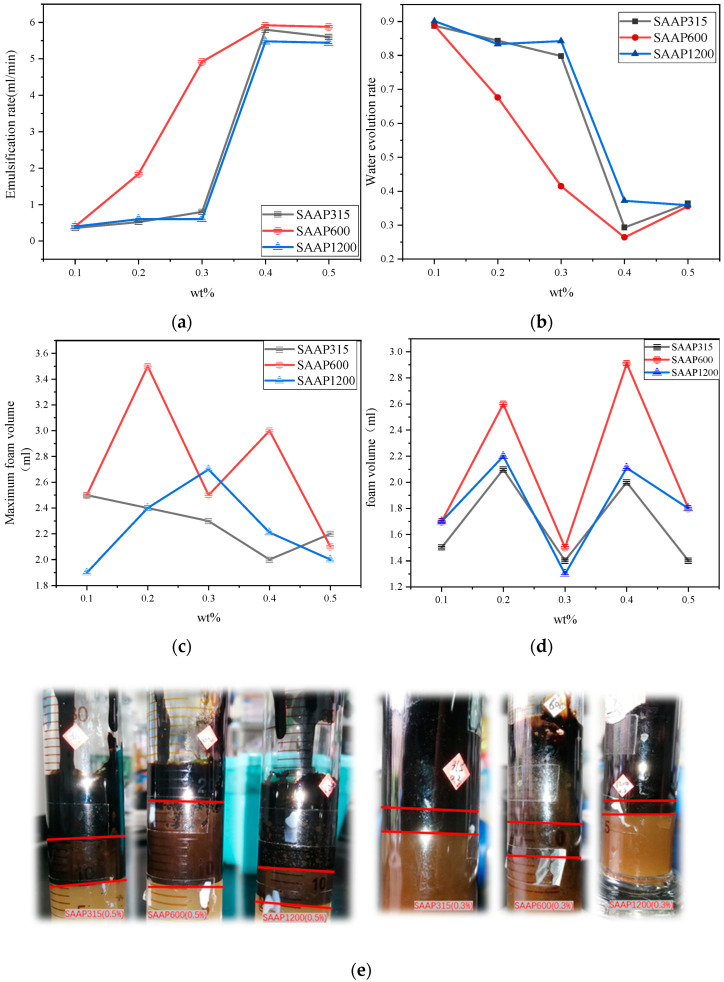
(**a**) Emulsification rate of solutions with different contents of three different viscosity reducers. (**b**) Water precipitation rate of emulsion formed by a viscosity-reducer solution with heavy oil at different contents. (**c**)Maximum foaming volume of different viscosity reducers at different mass fractions; (**d**) 5 min foam volume of different viscosity reducers at different mass fractions. (**e**) Physical diagram of different viscosity reducers with mass fractions of 0.3% and 0.5% forming an emulsion with heavy oil.

The emulsification speed of three different viscosity reducers on heavy oil at different mass fractions is shown in Figure 6a below. The emulsification speed of three viscosity reducers at different mass fractions was the highest for SAAP600. Moreover, the emulsification speed of three viscosity reducers increases with the increase of the mass fraction, and the rise of the emulsification speed is not apparent when it rises to 0.5%. This indicates that SAAP600 has the best emulsification performance. It is mainly because the SAAP600 molecule contains longer lipophilic side chains, and the number of side chains is also higher, which is conducive to SAAP600 adsorption on the surface of heavy oil particles. Then, through the sulfonic acid group and carboxylic acid group, which are two hydrophilic groups arranged on the outer side, the heavy oil particles and heavy oil particles are separated from each other by the water layer. It forms an O/W-type emulsion, which is not easy to gather [29]. For the heavy oil of equal volume, when the viscosity-reducer molecule is not yet saturated, the more content of the viscosity-reducer molecule, the more viscosity-reducer molecule is adsorbed on the surface of heavy-oil particles. Moreover, the heavy oil is easier to emulsify, and the emulsification speed undoubtedly increases. However, when the viscosity-reducer molecule reaches saturation, the heavy-oil particles can only adsorb so many viscosity-reducer molecules, and the remaining molecules are not adsorbed, so the emulsification speed reaches the upper limit and does not obviously increase. The emulsion formed by the 0.3% and 0.5% viscosity-reducer solutions and heavy oil are shown in Figure 6e below.

### 3.7. Stability of Different Polyether Carboxylate–Sulfonate Viscosity-Reducing Emulsions

The precipitation rate of emulsions formed by different content of viscosity-reducer solutions and heavy oil is shown in Figure 6b below. It can be seen from the graph that SAAP600 has the lowest water precipitation rate among the emulsions formed by three different mass fractions of viscosity-reducer solutions and heavy oil. This indicates that the emulsion formed by the corresponding viscosity reducers has the best stability. The water precipitation rate of the emulsion formed by three different mass fractions of viscosity reducers decreases with the increase of the mass fraction, which indicates that the stability of the emulsion formed by the three viscosity reducers and heavy oil is more stable with the rise of the mass fraction. This is because the molecules of the viscosity reducers are arranged in an orderly manner to form a molecular layer between the oil–water interface. Furthermore, the hydrophilic groups on the outside of the molecular layer are surrounded by water molecules through hydrogen bonding to create the first water molecular layer. The first water molecular layer then adsorbs the second water molecular layer through hydrogen bonding [23], which is the liquid film through which the emulsion disperses the heavy oil. The thickness of the liquid film is related to the length of the surfactant molecular chain, and the liquid film of giant molecule surfactants is thicker and more stable [30,31]. SAAP600 and SAAP1200 have significantly longer molecular weight lengths than SAAP315. In addition, because SAAP600 has more carboxyl and sulfonic acid groups than SAAP1200, its affinity with the water molecular layer is more robust, making the liquid film more stable, so the stability of SAAP600 to form an emulsion with heavy oil is better.

### 3.8. Foaming Performance and Foam Stability of Polyether Carboxylate–Sulfonate Viscosity Reducers

The foaming volume of three different viscosity reducers at different mass fractions and the foam volume after stabilization for 5 min are shown in Figure 6c,d below. According to the data graph, the foam volume of SAAP600 is the largest regardless of the maximum foam volume or the foam volume after stabilization for 5 min, which indicates that the foaming performance and foam stability of SAAP600 are better. When the surface tension is high, the solution cannot make it foam, and even if a small amount of foam is generated under the effect of oscillation, the foam cannot be kept stable; it will burst immediately after it is generated. When the surface tension is lowered to 38.18 mN/m, the solution starts to produce a stable foam, which indicates that the surface tension is reduced. The surface free energy of the system is lowered, making it easier to foam, the lower surface tension favors the stability of the foam [32,33]. It can be seen that in the range of a mass fraction of 0.1% to 0.5%, the three viscosity-reducer solutions have fewer molecules of viscosity reducers and have not reached saturation, when the molecules of viscosity reducers can all be arranged on the surface of the water. The longer side chains of SAAP600 do not interfere with each other, and the interference of the long side chains of SAAP1200 is reduced. At the same time, the number of molecules of SAAP315, which has the highest average molecular weight, is lower than the other two, and its ability to reduce surface tension is less than the other two, so the surface tension of SAAP600 solution is relatively low. However, when the viscosity reducer’s concentration is increased again, the data obtained should be similar to Figure 6b. After exceeding the critical micelle concentration, the reduction of SAAP315′s surface tension is more capable of doing so.

### 3.9. Optimization of Conditions for the Synthesis of Polyether Carboxylic Acid–Sulfonic Acid Viscosity Reducers

Among the three viscosity reducers, SAAP600, which had the best overall performance, was selected as the object of study to investigate the effects of monomer molar ratio, reaction time, and reaction temperature on the performance of the viscosity reducers.

#### 3.9.1. Effect of Monomer Molar Ratio

**Figure 7:**
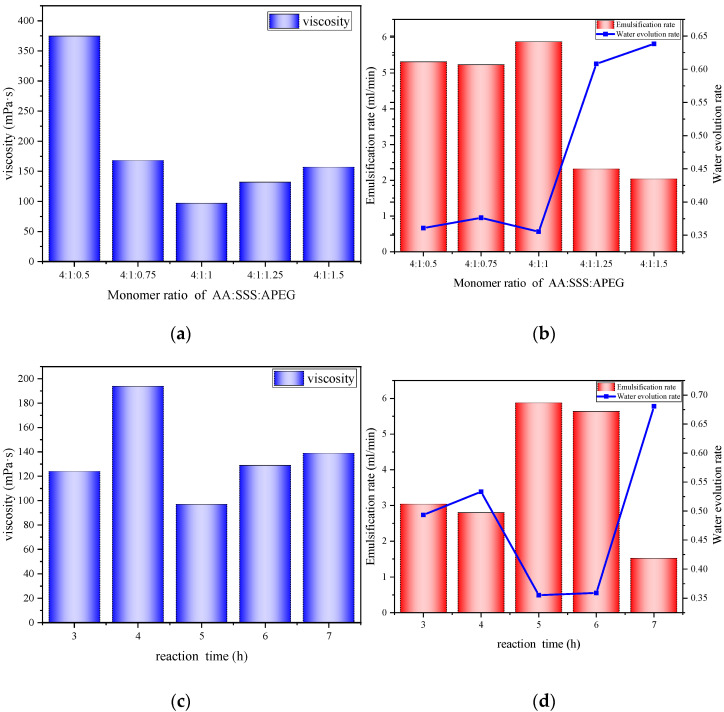

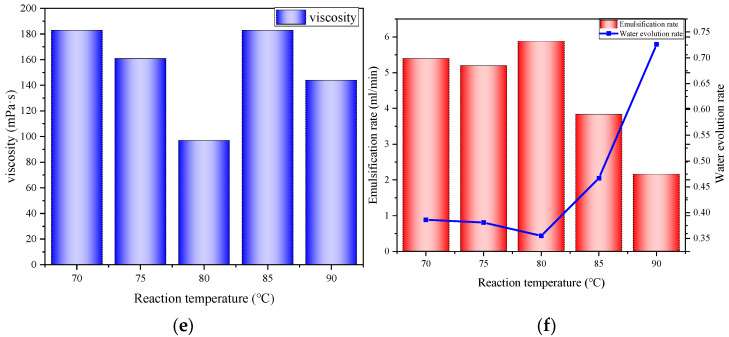
(**a**) Viscosity of emulsion formed by viscosity reducers with different monomer ratios and heavy oil. (**b**) Emulsification rate and water precipitation rate of viscosity reducers with different monomer ratios. (**c**) Viscosity of emulsion formed by viscosity reducers and heavy oil at different reaction times. (**d**) Emulsification rate and water precipitation rate of viscosity reducers at different reaction times. (**e**) Viscosity of viscosity reducers and heavy oil emulsions at different reaction temperatures. (**f**) Emulsification rate and water precipitation rate of viscosity reducers at different reaction temperatures.

With the other reaction conditions remaining unchanged, we changed the amount of APEG600, according to the molar ratio of AA/SSS/APEG600 = 4:1:0.5/0.75/1/1.25/1.5, respectively, to synthesize different viscosity reducers, and these viscosity reducers were tested and analyzed to determine their viscosity reduction performance and emulsification performance; the analysis results are shown in Figure 7a,b. According to the two figures, the viscosity reduction performance of five different proportions of viscosity reducers increases and then decreases with the rise of the proportion of APEG600, the emulsification speed increases and then decreases with the rise of the ratio, and the water precipitation rate decreases and then increases with the rise of the ratio. It can be seen that AA/SSS/APEG600 = 4:1:1 has the best viscosity reduction ability, the best emulsification performance, and the best stability of an emulsion. This is mainly because, as the ratio of APEG600 increases, the viscosity-reducer molecule contains more side chains, which is also beneficial to the adsorption of heavy oil, and the viscosity reduction performance and emulsification ability are enhanced. However, when the ratio of APEG600 is too large, the number of sulfonic acid groups and carboxylic acid groups on the viscosity-reducer molecule decreases, resulting in the viscosity-reducer molecule only able to adsorb on heavy oil. However, not enough adsorption capacity of water results in a thin liquid film, resulting in the emulsion stability becoming worse and the emulsification ability being reduced.

#### 3.9.2. Effect of Reaction Time

In the case of other reaction conditions remaining unchanged, according to the reaction time of 3 h, 4 h, 5 h, 6 h, and 7 h, respectively, five different viscosity reducers were synthesized. The viscosity reduction performance and emulsification performance of these viscosity reducers were tested and analyzed, and the analysis results are shown in Figure 7c,d. It can be seen from the figure that the viscosity reduction performance and emulsification performance of the viscosity-reducing agents with too short a reaction time are poor, and the emulsion formed is not stable enough. The main reason is that the reaction time is too short, and the monomer has not yet reacted completely. The molecular weight of the formed viscosity reducer is small, and the chain is short. Both the viscosity reduction performance and emulsification speed and stability are poor. The reaction time is too long. The monomer reaction is complete, and the viscosity reduction performance, emulsification speed, and stability are not much different. However, to save time and resources, the reaction time of 5 h is the best.

#### 3.9.3. Effect of Reaction Temperature

Under the condition that other reaction conditions remained unchanged, five different viscosity reducers were synthesized by reacting at the reaction temperatures of 70 °C, 75 °C, 80 °C, 85 °C, and 90 °C. The viscosity reduction performance and emulsification performance of these viscosity reducers were tested and analyzed, and the analysis results are shown in Figure 7e,f. It can be seen from the figure that the viscosity reduction performance and emulsification performance of the viscosity reducers reacting at 80 °C are the best, and the emulsion formation’s stability is also the highest. The reason is that at the beginning, as the reaction temperature increases, the rate of free radical reaction is accelerated, and the reaction is carried out thoroughly, so that the viscosity reduction performance and emulsification performance are improved. The reaction temperature is increased, which may exceed the applicable range of the initiator, resulting in the initiator not being able to trigger the reaction well, reducing the viscosity reduction performance, emulsification performance, and stability.

Finally, we concluded that the optimal reaction conditions for the synthesis of this polymeric surfactant were: 80 °C reaction temperature, 5 h reaction time, and a monomer ratio of AA/SSS/APEG600 = 4:1:1.

## 4. Conclusions

In this paper, a series of polyether carboxylic acid–sulfonic acid polymeric surfactants were synthesized. Through a series of tests, it was concluded that SAAP600 had the best viscosity reduction performance and that it could reduce the viscosity of heavy oil to 97 mPa·s. The best reaction conditions for the synthesis of this polymeric surfactant were a reaction temperature of 80 °C, a reaction time of 5 h, and a monomer ratio of AA/SSS/APEG600 = 4:1:1.

The mechanism of viscosity reduction was reasonably explained by testing the emulsification ability, wettability, and surface tension of the polyether carboxylic acid–sulfonic acid polymeric surfactant. In other words, by inserting the nonpolar lipophilic side chains into the heavy-oil particles, the asphaltene molecules initially distributed on the oil–water interface were replaced, and the net structure formed by the accumulation of asphaltene at the oil–water interface was destroyed. Meanwhile, the hydrophilic groups formed a water layer by mutual adsorption with water molecules through hydrogen bonding, and the stable water layer separated from the oil droplets into a more stable emulsion system, thus increasing the fluidity of heavy oil and decreasing the viscosity of heavy oil.

## Figures and Tables

**Table 1:** The relative molecular mass of viscosity reducers.

Viscosity Depressant	Mn	MW
SAAP315	8816	59,714
SAAP600	10,518	55,695
SAAP1200	10,103	46,576

**Table 2:** North China heavy oil main components table.

Main Components	Content
Pentadecane, 2,6,10,14-tetramethyl-	0.064342
Stigmastane	0.086701
beta.-iso-Methyl ionone	0.038009
Coprostane	0.036826
Cholestane	0.130264
Octadecane	0.0516781
Heptadecane	0.0583817
Tetracosane	0.0403763
Cholestan-3-one, 4,4-dimethyl-, (5.alpha.)-	0.0367927
Dodecane, 2,6,10-trimethyl-	0.0109083
Undecane	0.006227

## Data Availability

Not applicable.
